# Controllable fabrication and magnetic properties of double-shell cobalt oxides hollow particles

**DOI:** 10.1038/srep08737

**Published:** 2015-03-04

**Authors:** Dan Zhang, Jianyu Zhu, Ning Zhang, Tao Liu, Limiao Chen, Xiaohe Liu, Renzhi Ma, Haitao Zhang, Guanzhou Qiu

**Affiliations:** 1 School of Resources Processing and Bioengineering, Central South University, Changsha, Hunan 410083, PR China; 2 School of Materials Science and Engineering, Central South University, Changsha, Hunan 410083, PR China; 3 Institute of Process Engineering, Chinese Academy of Sciences, Beijing 100190, P. R. China

## Abstract

Double-shell cobalt monoxide (CoO) hollow particles were successfully synthesized by a facile and effective one-pot solution-based synthetic route. The inner architecture and outer structure of the double-shell CoO hollow particles could be readily created through controlling experimental parameters. A possible formation mechanism was proposed based on the experimental results. The current synthetic strategy has good prospects for the future production of other transition-metal oxides particles with hollow interior. Furthermore, double-shell cobalt oxide (Co_3_O_4_) hollow particles could also be obtained through calcinating corresponding CoO hollow particles. The magnetic measurements revealed double-shell CoO and Co_3_O_4_ hollow particles exhibit ferromagnetic and antiferromagnetic behaviour, respectively.

Multiple-shell hollow particles have attracted widely attention in recent years because of the promising properties and potential applications in various fields, such as lithium-ion batteries^1^,^2^,^3^,^4^, sensors^5^,^6^, photocatalysis^7^,^8^, dye-sensitized solar cells (DSSCs)^9^,^10^, drug/gene delivery^11^, microreactors^12^, and so forth. A variety of methods including template-assisted synthesis^13^, ionic exchange reaction^14^, Ostwald ripening^15^,^16^,^17^,^18^, Kirkendall effects^19^, and selective etching process^20^,^21^ have been developed to fabricate multiple-shell hollow particles. Among these methods, Ostwald ripening, a well-known physical phenomenon, which contains that the smaller size particles dissolving into the liquid phase as a nutrient supply for the growth of larger crystals and results in the formation of the hollow interior spaces, is one of the most effective approaches to the rational design of complex hollow structures. Up to now, remarkable progress has been made for the fabrication of multiple-shell hollow structures by Ostwald ripening process. In particular, wang and co-workers demonstrated a multistep Ostwald ripening approach for the geometry-controlled fabrication of Cu_2_O particles with multilayered shell-in-shell interior structures^22^. Zeng and co-workers reported the synthesis of double-shell SiO_2_ hollow spheres via Ostwald ripening process under solvothermal conditions^23^. Very recently, a family of multiple-shell structures, (Cu_2_O@)nCu_2_O (n = 1–4), has been synthesized through Ostwald ripening treatment at room temperature^24^. Despite these successes, developing the Ostwald ripening methods for the fabrication of multiple-shell hollow particles remains a highly sophisticated challenge.

Cobalt oxides have drawn increasing attention in the past decades on the basis of their distinctive electronic, magnetic, and catalytic properties and wide variety of applications. It is well known that CoO and Co_3_O_4_ are two especially important forms among the various cobalt oxides based on their distinctive structural features and fascinating properties. Generally, CoO, crystallizing in the rocksalt (NaCl)-like structure, consists of two face-centered-cubic (fcc) sublattices of Co^2+^ and O^2−^ ions, while Co_3_O_4_ belongs to the spinel-like structure based on a cubic close packing array of oxide ions, in which Co^2+^ ions occupy the tetrahedral 8a sites and Co^3+^ ions occupy the octahedral 16 d sites^25^. Because of the unique morphology-dependent properties, immense efforts have been dedicated to developing facile and effective approaches for the preparation of cobalt oxides with controllable morphologies, such as nanocone^26^,^27^, nanobelt^28^, nanoring^29^, nanocube^30^,^31^,^32^,^33^, nanowire^34^, nanotube^35^, hollow sphere^36^, etc. Recently, wang and co-workers have achieved a significant breakthrough in the synthesis of multiple-shell cobalt oxides hollow spheres by the use of carbonaceous microspheres (CMSs) as sacrificial templates^37^,^38^. Unfortunately, template-assisted synthesis generally involves tedious procedures including preparation of sacrificial templates, deposition of the designed materials, and selective removal of the templates via chemical etching or thermal decomposition. On the other hand, the removal of the CMSs templates may result in very low yield of target product based on the ion-absorption, which is unfavorable to fulfilling the application prospects of the multiple-shell hollow spheres.

Herein, we present a one-pot solvothermal method to prepare double-shell CoO hollow particles. Interestingly, the inner and outer architecture of the double-shell CoO hollow particles can be readily tuned through controlling experimental parameters. The results demonstrate that the double-shell hollow particles might form through Ostwald ripening. By using CoO hollow particles as the precursor, double-shell Co_3_O_4_ hollow particles can also be obtained via thermal decomposition process. Notably, the current synthetic strategy may provide an effective route for the synthesis of other transition-metal oxides particles, and is thus promising for achieving unique architectures with hollow interior for a wide range of applications.

## Results

**Figure 1:**
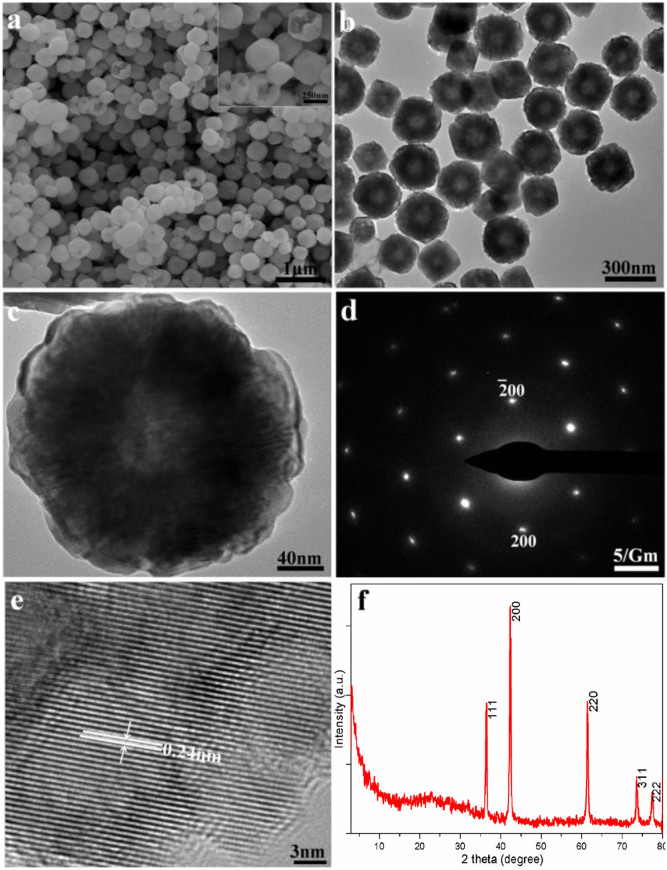
Solvothermal synthesis of double-shell CoO hollow particles obtained at 260°C for 8 h. (a) SEM and (b, c) TEM images. The inset in a) shows a higher magnification SEM image. (d) SAED pattern and (e) HRTEM image of the individual double-shell CoO hollow particle. (f) XRD pattern of as-prepared double-shell CoO hollow particles obtained at 260°C for 8 h.

Figure 1a displays a representative SEM image of as-prepared product obtained using cabalt (II) acetylacetonate as cobalt source at 260°C for 8 h, in which a large quantity of near-spherical particles with good uniformity were achieved under current conditions. The particles have a mean size of about 300 nm. There exist many broken particles, which reveals that CoO particles obviously possess hollow interiors. The inset is a high-magnification SEM image obtained from a selected area of Figure 1a. Herein, the hollow interiors of as-prepared CoO particles can be clearly identified. Figure 1b presents a typical TEM image of CoO particles, which also evidently exhibits CoO particles with hollow interiors. Figure 1c indicates a typical TEM image of an individual CoO hollow particle. With careful observation, the double-shell structure of CoO hollow particle can be clearly observed. The outer and inner shell thicknesses of the double-shell CoO hollow particle are estimated to be about 8 and 100 nm, respectively. A selected area electron diffraction (SAED) pattern taken from the individual CoO hollow particles, as shown in Figure 1d, illustrates single-crystalline structure of double-shell CoO hollow particles. Figure 1e depicts the HRTEM image of the individual particle. The lattice spacing is calculated to be about 0.24 nm, agreeing well with the value of {111} lattice planes of cubic CoO. The crystal structures of as-prepared products were characterized by X-ray powder diffraction (XRD). All of the reflections of the XRD pattern, as shown in Figure 1f, can be readily indexed as a face-centered cubic phase of CoO (JCPDS 65–2902) with lattice constant *a* = 0.426 nm (space group: Fm-3m (No. 225)). No impurity peak was observed, indicating the high purity of the product obtained under such conditions. The sharp diffraction peaks also reflects the good crystallinity of as-prepared product.

**Figure 2:**
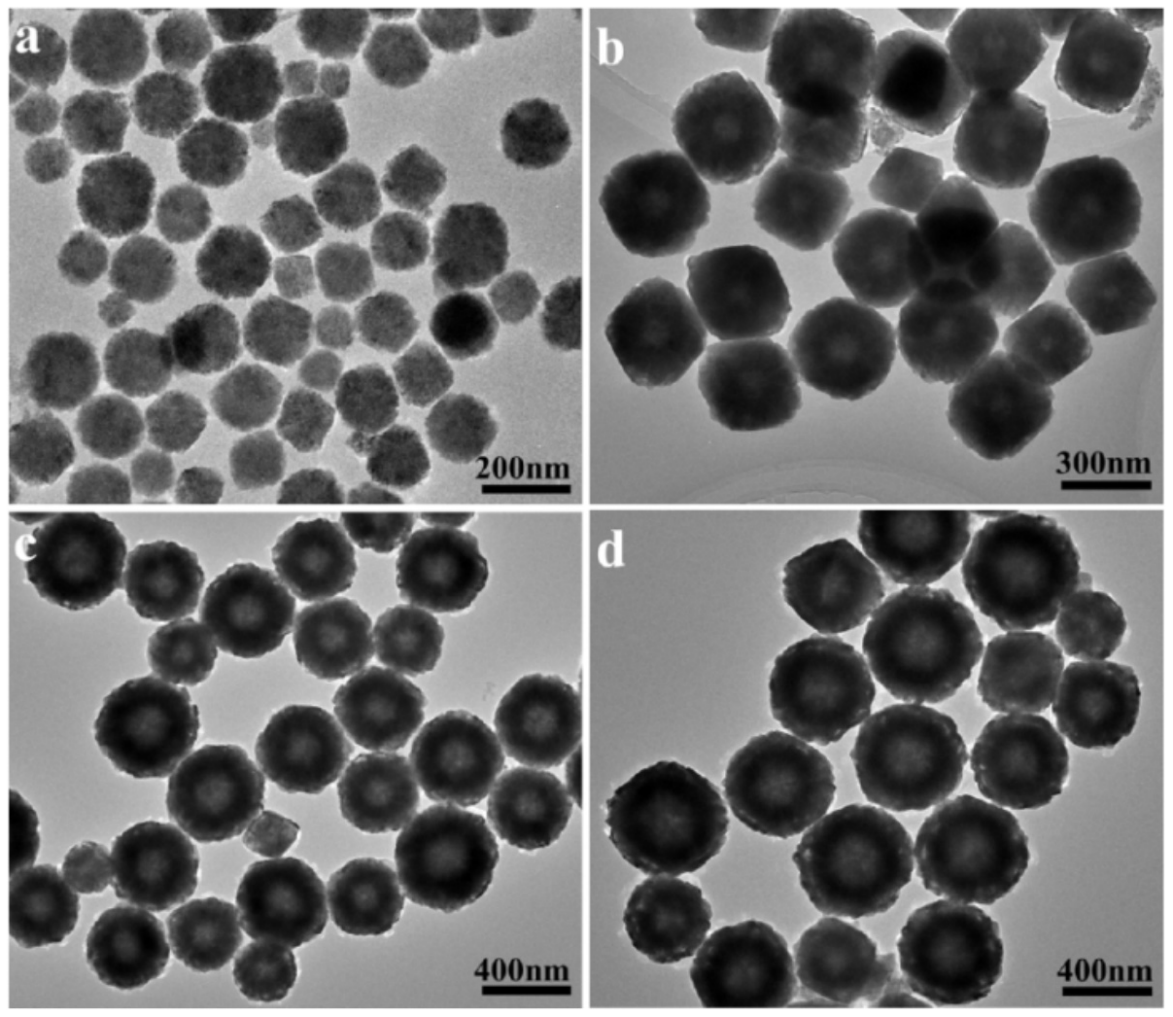
TEM images of CoO particles prepared at 260°C for varied time durations: (a) 1 h, (b) 4 h, (c) 12 h, and (d) 24 h.

For a better understanding of the growth process of double-shell hollow particles, the influences of reaction time on the morphologies of products have been investigated. Figure 2 summarizes a series of morphological observations supposed to be in different stages of forming double-shell hollow particles. When the reaction time was decreased to 1 h, the product is mostly made up of near-spherical solid particles with average size of about 150 nm, as shown in Figure 2a. Figure 2b depicts a typical TEM image of product obtained for 4 h. The mean size of CoO particles is increased to about 250 nm, suggesting the elongation in size with increasing reaction time. Closer observation reveals that almost all particles possesses hollow interiors at this stage. Extending the reaction time to 12 h, the double-shell structures of the CoO hollow particles with sizes in the range 300–400 nm can be observed more clearly (Figure 2c). It is noteworthy that if the reaction time is further extended to 24 h, almost 100% double-shell CoO hollow particles with uniform sizes about 500 nm can be obtained, as shown in Figure 2d. In particular, the outer shell thickness of CoO hollow particle is increased to about 45 nm, comparable to that of CoO hollow particles obtained for 12 h.

**Figure 3:**
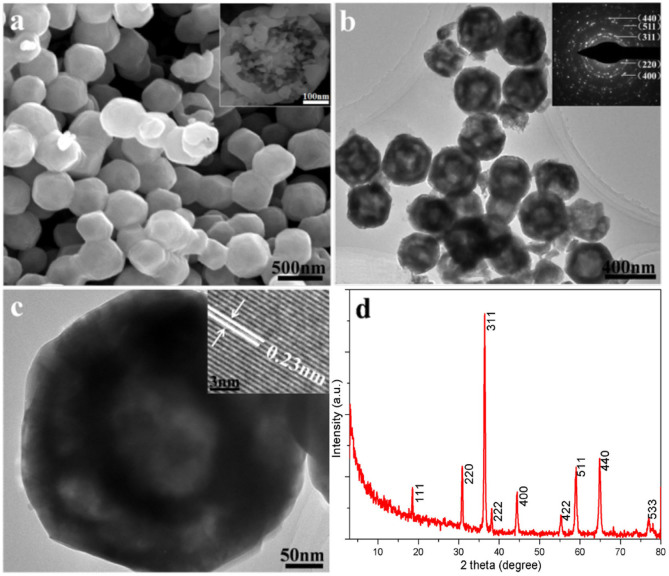
(a) SEM image of double-shell Co_3_O_4_ hollow particles obtained by calcination of as-prepared CoO hollow particles at 600°C for 2 h in air. The inset shows an individual Co_3_O_4_ hollow particle. (b) Low- and (c) high-magnification TEM images of double-shell Co_3_O_4_ hollow particles. The insets in (b) and (c) show SAED pattern and HRTEM image of double-shell Co_3_O_4_ hollow particles, respectively. (d) XRD pattern of as-prepared double-shell Co_3_O_4_ hollow particles.

Double-shelled Co_3_O_4_ hollow particles could be also successfully obtained via calcination method using corresponding CoO hollow particles obtained at 260°C for 8 h as precursors. Figure 3a depicts the SEM image of as-prepared Co_3_O_4_ obtained by calcination of corresponding CoO hollow particles at 600°C for 2 h in air. Compared with CoO hollow particles, the average size of as-prepared Co_3_O_4_ is estimated to be about 400 nm, which may be related to the possible oxidation of CoO to Co_3_O_4_. An individual particles with broken shell shown in the inset of Figure 3a demonstrates the ball-in-ball structure of the Co_3_O_4_ hollow particles. A typical TEM image of as-prepared Co_3_O_4_ hollow particles is shown in Figure 3b. Herein, the Co_3_O_4_ hollow particles with double-shell structure can be clearly observed. In the inset, an SAED pattern was well indexed to spinel Co_3_O_4_, revealing a polycrystalline nature of the calcined hollow particles. The double-shell structure is also clearly revealed by TEM observation of an individual Co_3_O_4_ hollow particle, as shown in Figure 3c. The inset displays the corresponding HRTEM image, which provides further insight into the structure of Co_3_O_4_ hollow particle. Lattice spacing is measured to be about 0.23 nm, which is consistent with the interplanar spacings of {222} for spinel Co_3_O_4_. Figure 3d shows the typical XRD pattern of double-shell Co_3_O_4_ hollow particles. All the reflections in the XRD pattern can be indexed as a face-centered cubic phase of spinel Co_3_O_4_ (JCPDS 43-1003) with lattice constant a = 0.808 nm (space group: Fd-3m (No. 227)). No impurity peaks were observed, indicating that cubic CoO was completely converted into spinel Co_3_O_4_.

**Figure 4:**
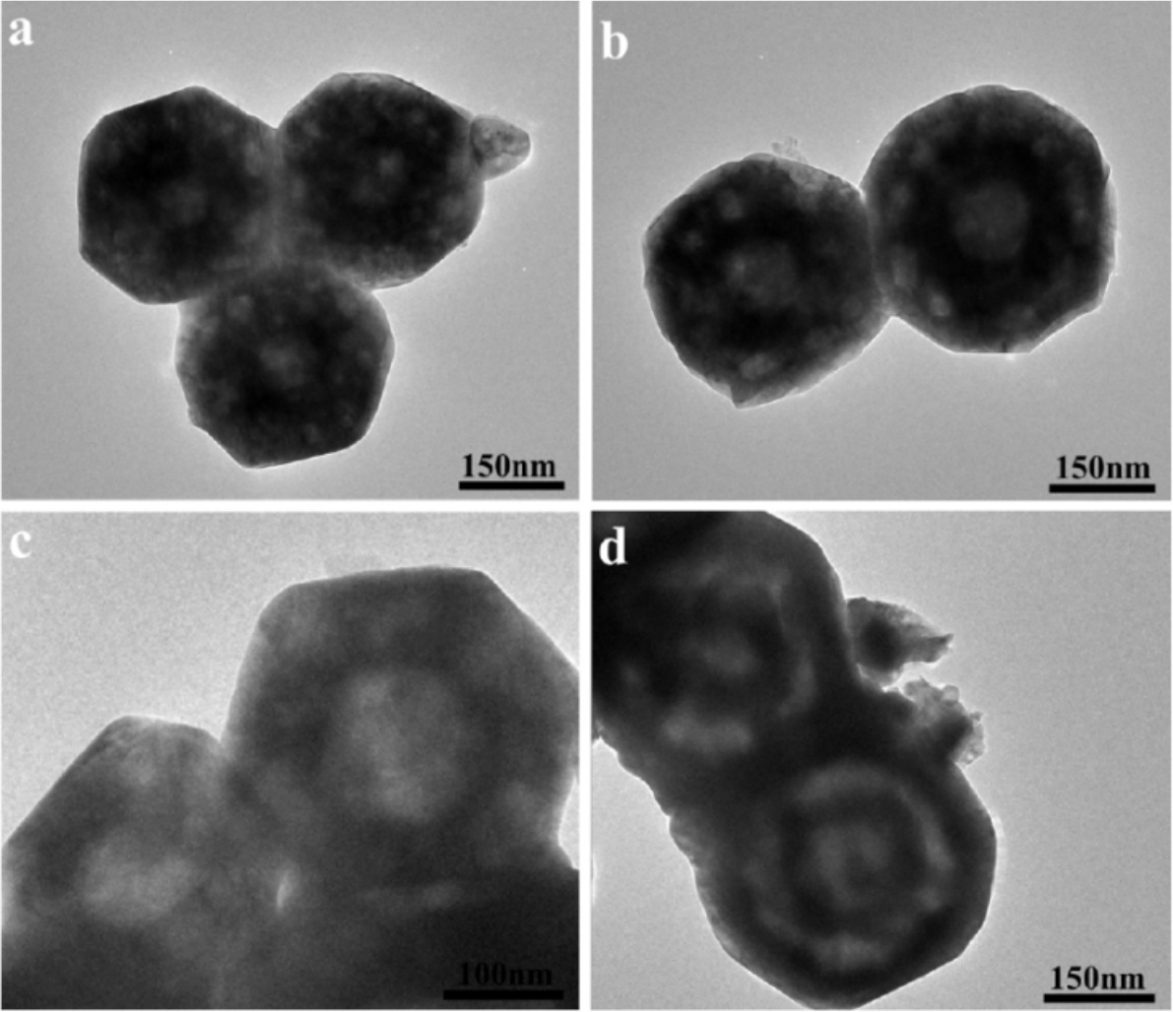
TEM images exhibit the formation stages of double-shell Co_3_O_4_ hollow particles prepared by calcination of CoO particles as the precursor obtained at 260°C for varied time durations: (a) 2 h, (b) 4 h, (c) 6 h, and (d) 12 h.

The evolution of double-shell structure during the calcination of as-prepared CoO particles obtained at 260°C for varied time durations as the precursor at 600°C for 2 h directly mirrors a possible scenario for the formation of double-shell Co_3_O_4_ hollow particles. Figure 4 summarizes a series of morphological observations of forming double-shell Co_3_O_4_ hollow particles. Figure 4a shows a typical TEM image of the porous Co_3_O_4_ with hollow interior prepared by calcination of corresponding CoO particles obtained at 260°C for 2 h. With careful observation, the shells of Co_3_O_4_ hollow particles were found to be composed of many pores with a mean diameter of 15 nm. It is noteworthy that if the reaction time of precursors was extended to 4 h, large interior space of Co_3_O_4_ hollow particles can be generated, as shown in Figure 4b. Typical TEM image shown in Figure 4c indicates a significant increase of pore sizes in the shells. Furthermore, the interior space is also further improved. This suggests both the expansion in interior space and the addition in pore size with increasing reaction time. More interestingly, Co_3_O_4_ hollow particles with apparent double-shell structure can be clearly identified using CoO hollow particles as precursors obtained at 260°C for 8 and 12 h, as shown in Figure 3 and 4d, respectively.

## Discussion

**Figure 5:**
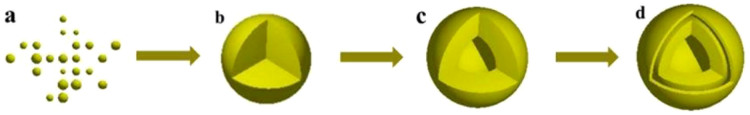
Schematic exhibition of the formation of double-shell CoO hollow particles.

To investigate the mechanism of double-shell CoO hollow particles, based on the experimental results, the possible scenario for the formation of double-shell CoO hollow particles is illustrated in Figure 5. Firstly, Co(acac)_2_ dissolves in organic solvent and then undergoes thermal decomposition to form small particles under solvothermal conditions. Subsequently the fresh small particles is unstable due to the high surface energy and tends to aggregate into larger solid particles driven by the minimization of interfacial energy, which can then convert into hollow particles due to the Ostwald ripening process^39^. Finally, owing to the existence of anisotropic outer surfaces of the hollow particles, hollowing space gradually takes place at a particular region underneath the outer surface and leads to the formation of double-shelled hollow structures^40^,^41^.

**Figure 6:**
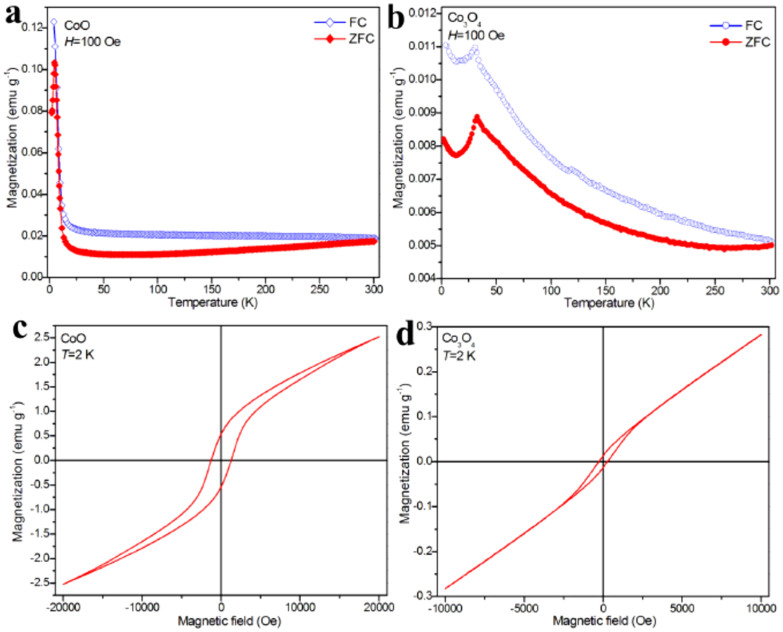
ZFC and FC magnetization curves for (a) CoO and (b) Co_3_O_4_ hollow particles measured under an applied field of 100 Oe. Isothermal magnetization curves for (c) CoO and (d) Co_3_O_4_ hollow particles at 2 K.

The magnetic properties of double-shell cobalt oxides hollow particles were measured on a superconducting quantum interference device (SQUID). The temperature dependences of the zero-field-cooled (ZFC) and field-cooled (FC) magnetization of the double-shell CoO hollow particles measured under an applied field of 100 Oe are shown in Figure 6a. It is clear that there is a significant difference between ZFC and FC curves of double-shell CoO hollow particles at low temperature. Compared with FC curve, the ZFC curve depicts a distinct peak at 4.8 K, suggesting ferromagnetic (FM) behavior below 4.8 K, which may be attributed to superparamagnetic cobalt particles^42^. Figure 6b shows the ZFC and FC curves of double-shell Co_3_O_4_ hollow particles measured under an applied field of 100 Oe. The feature indicates the possible presence of antiferromagnetic (AFM) transitions. The AFM transition occurs at ~32 K (Néel temperature, *T*_N_), being far lower than that of the bulk Co_3_O_4_ known at about 40 K, which is possibly resulted from the finite size and surface effect of double-shell Co_3_O_4_ hollow particles^43^,^44^. The hysteresis loops for double-shell CoO and Co_3_O_4_ hollow particles at 2 K are shown in Figure 6c and d, respectively. The coercivity value *H*_c_ for the CoO is about 1298 Oe at 2 K, indicating the presence of ferromagnetic ordering component. For Co_3_O_4_ hollow particles, despite of that the low temperature data show a slight curvature, the magnetization curves are nearly linear at 2 K, also indicative an antiferromagnetic ground state.

## Methods

The synthesis use commercially available reagents: cabalt (II) acetylacetonate (Co(acac)_2_, chemical grade, 98%, a&k), 1-octadecene (ODE, technical grade, 90%, ACROS), oleic acid (OA, chemical grade, SCRC), and oleylamine (OAm, technical grade, approximate C18-content 80–90%, ACROS) were of chemically pure and used as received.

### Preparation of double-shell CoO hollow particles

Typical synthetic procedures are summarized as follows: 0.2571 g Co(acac)_2_ (1 mmol) was dissolved into 20 mL ODE with OA and OAm (in molar ratios of 4:10) to form colloid mixture under vigorous stirring for 10 min at room temperature. Then the mixture was sealed in a Teflon-lined and maintained at 260°C for 1–24 h. The autoclave was cooled to room temperature. The brown products were washed for several times with absolute ethanol and hexane. Finally, the products were dried in vacuum at 60°C for 6 h.

### Preparation of double-shell Co_3_O_4_ hollow particles

The double-shell Co_3_O_4_ hollow particles could be prepared by the thermal decomposition of as-prepared CoO hollow particles obtained at 260°C for 2–12 h as precursors calcinated at 600°C for 2 h in air.

### Characterization

The structure and phase composition of the products were characterized on a X-ray diffractometer (XRD, D/max2550 VB+) with Cu Kα radiation (λ = 1.5418 Å). The morphologies and sizes of the products were characterized by a field-emission scanning electron microscopy (FE-SEM, Sirion 200) and transmission electron microscope (TEM, Tecnai G2 F20). High-resolution TEM (HRTEM) images and SAED patterns were obtained from the TEM. Magnetic measurements were conducted using a Quantum Design MPMS XP-5 superconducting quantum interference device (SQUID).

## Author Contributions

D.Z., J.Z., X.L. and G.Q. conceived and designed the experiments, and X.L. and G.Q. supervised the research; N.Z., T.L. and L.C. helped to synthesize the hollow spheres; R.M. and H.Z. assisted in the SQUID studies; D.Z. performed the synthesis and characterization, interpreted the data and wrote the paper with help from X.L. and G.Q.
